# Reference-Free Comparative Genomics of 174 Chloroplasts

**DOI:** 10.1371/journal.pone.0048995

**Published:** 2012-11-20

**Authors:** Chai-Shian Kua, Jue Ruan, John Harting, Cheng-Xi Ye, Matthew R. Helmus, Jun Yu, Charles H. Cannon

**Affiliations:** 1 Key Laboratory of Tropical Forest Ecology, Xishuangbanna Tropical Botanic Garden, Chinese Academy of Sciences, Menglun, Yunnan, People's Republic of China; 2 Graduate School of the Chinese Academy of Sciences, Beijing, People's Republic of China; 3 The CAS Key Laboratory of Genome Science and Information, Beijing Institute of Genomics, Chinese Academy of Sciences, Beijing, People's Republic of China; 4 Department of Biological Sciences, Texas Tech University, Lubbock, Texas, United States of America; 5 Department of Computer Science and Center for Bioinformatics and Computational Biology, Institute for Advanced Computer Studies, University of Maryland, College Park, Maryland, United States of America; 6 Amsterdam Global Change Institute, Vrije Universiteit, Amsterdam, The Netherlands; University of Arizona, United States of America

## Abstract

Direct analysis of unassembled genomic data could greatly increase the power of short read DNA sequencing technologies and allow comparative genomics of organisms without a completed reference available. Here, we compare 174 chloroplasts by analyzing the taxanomic distribution of short kmers across genomes [1]. We then assemble *de novo* contigs centered on informative variation. The localized *de novo* contigs can be separated into two major classes: tip = unique to a single genome and group = shared by a subset of genomes. Prior to assembly, we found that ∼18% of the chloroplast was duplicated in the inverted repeat (IR) region across a four-fold difference in genome sizes, from a highly reduced parasitic orchid [2] to a massive algal chloroplast [3], including gnetophytes [4] and cycads [5]. The conservation of this ratio between single copy and duplicated sequence was basal among green plants, independent of photosynthesis and mechanism of genome size change, and different in gymnosperms and lower plants. Major lineages in the angiosperm clade differed in the pattern of shared kmers and *de novo* contigs. For example, parasitic plants demonstrated an expected accelerated overall rate of evolution, while the hemi-parasitic genomes contained a great deal more novel sequence than holo-parasitic plants, suggesting different mechanisms at different stages of genomic contraction. Additionally, the legumes are diverging more quickly and in different ways than other major families. Small duplicated fragments of the *rrn*23 genes were deeply conserved among seed plants, including among several species without the IR regions, indicating a crucial functional role of this duplication. Localized *de novo* assembly of informative kmers greatly reduces the complexity of large comparative analyses by confining the analysis to a small partition of data and genomes relevant to the specific question, allowing direct analysis of next-gen sequence data from previously unstudied genomes and rapid discovery of informative candidate regions.

## Introduction

### Comparative genomics in the next-gen sequencing era

Technological advances in genomic sequencing have made it possible to acquire vast amounts of DNA sequence data for any organism quickly and cheaply [6]. The short-read genomic sequencing technology was originally intended for re-sequencing model organisms with completed reference genomes available [7]. For biologists working on non-model organisms without a reference genome, the *de novo* assembly of newly sequenced genomes and their comparative analysis is considerably more complicated and difficult. Accurate and full *de novo* assembly requires prodigious data coverage, the construction of numerous libraries, and extensive finishing of the genome assembly [8], both of which are frequently beyond the scope, budget, and requirements of ecological or evolutionary studies of non-model organisms. While partial assembly can provide informative markers [9], a large fraction of the available genomic data remains unanalyzed. For most comparative questions in ecology and evolution, the portion of the genome relevant to the answer is typically small, therefore the challenge lies in discovering these informative regions efficiently and prior to significant investment in *de novo* assembly. Direct analysis of next-gen genomic sequence data could greatly simplify large comparative studies.

**Figure 1:**
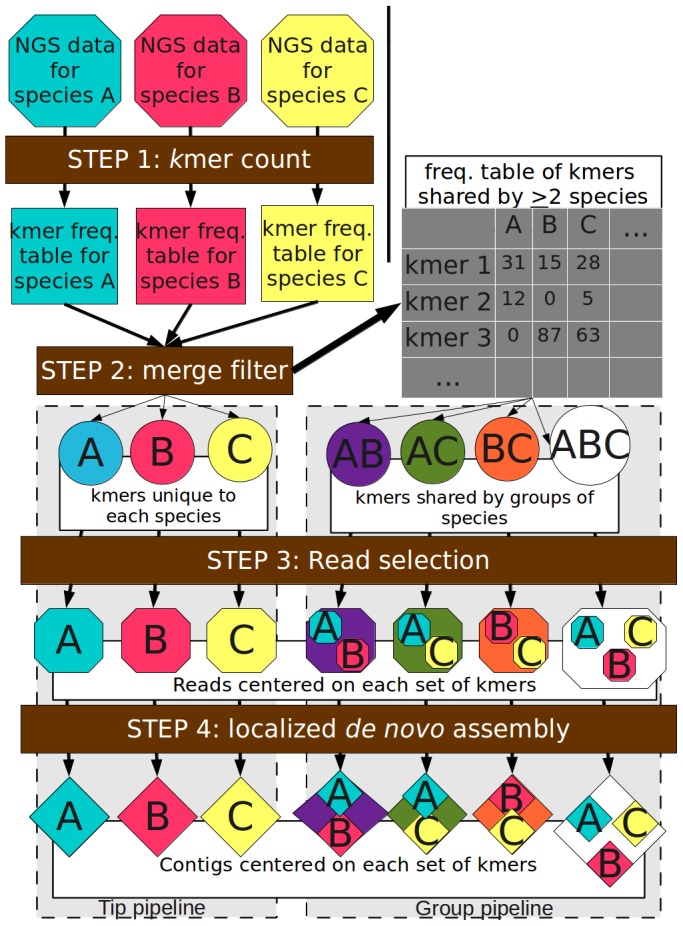
Flowchart of reference-free comparative genomic analysis. Step 1:SRS data from each sample is converted into a *kmer* frequency table, using any *k*mer counter. Step 2: *k*mers unique to a single genome are filtered into separate subsets while *k*mers shared by at least two genomes are merged into a single frequency table. Step 3: The SRS reads containing the kmers for both the unique and shared groups of kmers are clustered into separate subsets, keeping the reads from each genome in a group separate. Step 4: localized *de novo* contigs for each genome in each subset are assembled. ‘Tip’ contigs are assembled from kmers unique to each genome while ‘group’ contigs are assembled from kmers shared by at least two genomes in separate pipelines as indicated by the dashed vertical line.

Here, we present a reference-free comparative genomic approach (Fig. 1) that performs the comparative analysis prior to assembly, characterizing basic properties and segregating nucleotide sequence variation into smaller data partitions according to its distribution across genomes. Subsequent *de novo* assembly is therefore confined to only the portion of the genomic data relevant to a specific comparative question. The approach can also identify portions of the genomic data that contain informative variation but are recalcitrant to assembly. Our approach is similar to the DIAL pipeline [10] but our approach is considerably more general in its application: it detects all sequence variants, including translocations and insertions (see Methods), in addition to SNPs; it identifies regions with a high density of informative sequence variation; it simultaneously compares numerous genomes of any phylogenetic relatedness; and it segregates sequence variation according to the genomes which share that variation.

### Analysis of 174 chloroplast genomes

The chloroplast genome is the most comprehensively studied plant genome [11]. Because of its unique molecular structure and its uni-parental inheritance, the chloroplast genome has many excellent properties for evolutionary analysis. To demonstrate the effectiveness of our approach, we compare 174 complete chloroplast genomes (see Table S1 for list of for complete list of NCBI accession numbers and taxonomy), encompassing a wide range of taxonomic groups and representing both deep phylogenetic splits and closely-related clusters of species in the same genus. While the approach is intended to start without the benefit of a reference genome, we perform this analysis using already completed genomes to validate its effectiveness and power. Accordingly, we simulated a sequencing run of each chloroplast genome to produce Illumina-like short-read sequences of 51 bp with approximately 20× coverage [12]. Two data sets were simulated, one with no error whereas another using a 5% error model.

The chloroplast genome is remarkably conserved in its molecular structure and function among all green plants, as the size range (∼60–200 kb for Viridiplantae) is narrow compared to plant mitochondria. The basic structure of the circular genome is largely conserved from algae through the asterids, equivalent to almost one billion years of divergence [13]. Most land plants and algae chloroplasts contain a large (LSC) and a small (SSC) single copy genic region, consisting of numerous key functional genes, separated by two copies of inverted repeat regions (IRA and IRB). The exact function of the IR regions is unclear but it is not essential to chloroplast function, as numerous species have lost substantial portions of these duplicated regions, including in all sequenced species of the pines [14] and numerous legume species that form an “Inverted Repeat Lacking Clade” [15], [16]. Additionally, holo-parasitic plants, which have completely lost their photosynthetic abilities, retain their chloroplast genome, which generally becomes reduced in size [2], [17]–[19]. Examining the breakdown of structure and function in these vestigial organelles has also been informative to our understanding of chloroplast function [2].

Given the large number of questions that could be addressed among these 174 chloroplasts, we focus on a small set of comparative results in this publication, using the updated taxonomy provided by NCBI to provide our phylogenetic framework (see Table S1 for taxonomy). Results for tip contigs for all the studied groups are provided as supplementary information (see Tables S2, S3). Other sets of contigs can be provided upon request. We also provide open-access and generalized C and python-based scripts for other users to run the comparative analysis (see Methods). We first examine basic properties of *k*mer diversity in these genomes. Secondly, we examine the ‘tip’ contigs or the assemblies. The ‘tip’ portion of the pipeline (Fig. 1) is based upon the *k*mers unique to each genome. As mentioned above, several important economic plant families, like mustards (Brassicaceae), legumes (Fabaceae), and grasses (Poaceae), are represented by numerous genera, while most lower plants, like liverworts, are represented of a few deeply divergent species. This range of phylogenetic distance among genomes will provide considerable insight into the types of sequences that are unique in a particular genome given its relatedness to other genomes in the analysis. Finally, we examine the ‘group’ contigs. The ‘group’ portion of the pipeline (Fig. 1) is based upon the *k*mers shared by two or more species. Again, because the number of possible groups of genomes is large, we only address two major comparative questions: 1) what chloroplast regions are deeply conserved among the Viridiplantae; and 2) does chloroplast evolution differ among the four species-rich families (the legumes, the grasses, the mustards, and the pines)?

## Results and Discussion

### *K*mer diversity and genome size

Because we observed little difference in the results with and without sequencing error, we only present the results using simulated data with error. Additionally, sequencing error actually increased the phylogenetic signal in the size distribution of contigs (Figure S1). The simulated error rate (5%) increased the proportion of longer contigs among each set of localized *de novo* contigs. We examined whether sequencing error affected assembly accuracy by aligning a random subset of tip contigs against their reference and found that error had a very limited effect on accuracy (>97% of 366 contigs without error and >96% of 1964 contigs with error were identical to their reference). All contigs were within 99% identity of their reference. For data with error, the false positive rate was only 10% for “single feature” contigs (∼90–110 base pairs with 51 bp reads), each containing a single nucleotide variant. More detailed analysis suggested that random error in the short read sequence data acts as a weak ‘linker’ between informative regions where variants are less than read length apart, increasing the probability that short contigs would be combined into a longer contig by pulling in the reads between the variants. This error is not incorporated into the contigs because the reads containing the error are typically at a much lower frequency than the correct sequence, so the error is corrected from the assembled contig. Therefore, our approach copes well with moderate levels of sequencing error but this error actually increased the informativeness of the results, allowing us to identity hot-spots of diversification. Since real DNA sequence data always has error, subsequent discussion will focus on the results from the data with error.

**Figure 2:**
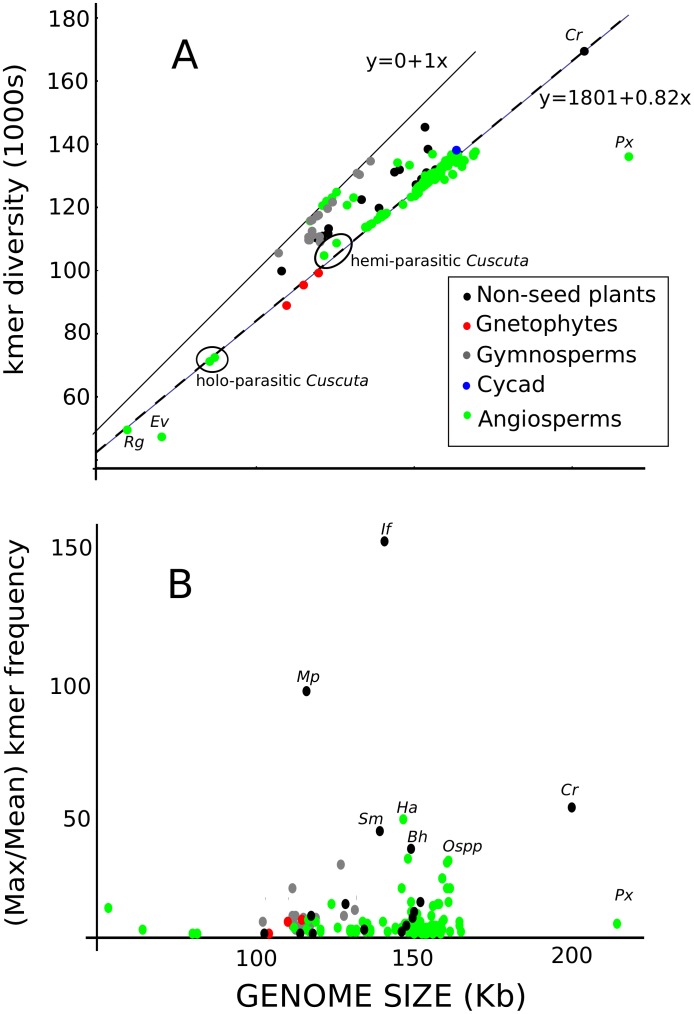
Relationship between genome size and kmer diversity (k = 21) among 174 chloroplasts. The solid black line indicates a 1∶1 ratio between genome size and kmer diversity while the blue dashed line is fit to the chloroplasts with inverted repeats (kmers = 1801+0.82*genome size). A) 174 chloroplasts, color-coded as indicated in the legend. Several taxa are indicated by the following codes: *Rg* = *Rhizanthella gardneri*; *Ev* = *Epifagus virginiana*; Cr = *Chlamydomonas reinhardtii*; *Px* = *Pelargonium*_*x*. The two pairs of *Cuscuta* taxa are indicated by ellipses, according to whether they are holo- or hemi-parasitic. B) Angiosperm chloroplasts 110–170 kb, color-coded as in the legend. Several taxa are indicated by the following codes: *Ce* = *Cuscuta exaltata*; *Ms* = *Monsonia speciosa*; *Et* = *Erodium texanum*; *Ts* = *Trifolium subterraneum*; *Io* = *Illicium oligandrum*; *Gp* = *Geranium palmatum*.

Optimal *k* length depends on many factors, particularly the relatedness of the compared genomes and probably needs to be optimized for every assembly, including combinations of k values. Because our analysis encompasses a very large phylogenetic distance, we present only the results for *k* = 21 base pairs. We examined the preliminary results for a range of *k* values (17–31 bp), the broader patterns were the same for most values of *k* while the extremes (17 and 31 bp) were least informative. The *k*mer frequency tables for each individual species (Fig. 1 – step 1) ranged between 58,940 unique *k*mers in *Epifagus virginiana* to 178,853 unique *k*mers in *Chlamydomonas reinhardtii* (see Table S1). Species without an inverted repeat (IR) region had an almost 1∶1 ratio between chloroplast size and *k*mer diversity (Fig. 2A), indicating little sequence duplication in these genomes. These plants include several legumes (Fabaceae) and gymnosperms of several families including Pinaceae and Cupressaceae.

Among taxa with a recognizable IR region, a strong correlation existed between genome size and *k*mer diversity (number of unique *k*mers = 1801+0.82 * genome size in bp). This relationship (Fig. 2A) is true across a four-fold difference in genome size (from 59 kb in a holo-parasitic orchid, *Rhizanthella gardneri*, to 204 kb in a single cell alga, *Chlamydomonas reinhardtii*) and despite a divergence time of almost one billion years and complete loss of photosynthetic function. This result indicates that roughly 18% of the chloroplast genome is duplicated in the IR regions in the vast majority of green plants and this ratio between single and double copy DNA is remarkably stable, given a wide range of evolutionary conditions.. Several non-seed plants and conifers, on the other hand, were considerably more variable in this relationship but only two species, the tiny holo-parasitic *Epifagus virginiana* [19] and the greatly expanded *Pelargonium*×*hortorum* [20], possessed substantially greater fractions of duplicated sequence in their chloroplast genomes. The 18% duplication rate appears to be a strong upper bound for almost all chloroplast genomes. Towards the upper end of genome size, several flowering plant species exhibited slightly higher rates of duplication, including three asterids: *Trachelium caeruleum* (Blue throatwort), *Jasminum nudiflorum* (Winter jasmine), and *Ipomoea purpurea* (Common morning glory); and all five species of evening primrose in the genus *Oenothera*, all of which have been noted to have high fractions of repetitive elements [21]–[23].

Two examples of substantial genome expansion existed in the analysis. While *Chlamydomonas*, a unicellular alga with a 204 kbp chloroplast genome fits the general trend, the flowering plant *Pelargonium* (Geraniaceae) with a 218 kbp genome falls far below the line, indicating a very substantial amount of overall duplication. Surprisingly, the most frequent *k*mers in these two genomes are dramatically different, despite the substantial expansion in number of repeats in both. The most frequent *k*mers in the algal genome are over 50× as frequent as the mean *k*mer frequency, while the most frequent kmer in pelargonium is only 5× as frequent as the mean frequency (Fig. 2B). This stark difference suggests a different process of genome expansion between the two taxa. Very short repetitive elements seem to have proliferated in the algal genome [3] while much larger pieces of nucleotide sequences were duplicated in the flowering plant [20]. The proliferation of very short repetitive elements has also occurred in several seedless plants, particularly *Isoetes*, the quillwort fern-ally, where the most frequent *k*mer is 150× the mean (Fig. 2B). Very few flowering plants exhibited this kind of very short element proliferation, except *Helianthus* and *Parthenium* (both in Asteraceae) and the several species of *Oenothera* (Onagraceae), suggesting that mechanisms responsible for this proliferation of very short repeats were lost in the ancestral seed plant, although it re-appears in a few lineages.

Conspicuous among angiosperms, all five species from four genera in the family Geraniaceae (see *Px* in Fig. 2A and *Ms*, *Et*, and *Gp* in Figure S2) deviated from the general trend, further evidence that chloroplast evolution is highly unusual in this group [20], [24]. While *Pelargonium* has an IR region three times the normal size at 76 kb, the IR region has been lost completely in the two *Erodium* species and reduced in *Geranium palmatum* (11 kb) and *Monsonia speciosa* (7 kb). The four latter species still possess an intermediate level of duplication in the chloroplast genome, despite the highly reduced IR regions, without any dramatic increase in the frequency of *k*mers, agreeing with previous results indicating that families of large dispersed repeats associated with rearrangement endpoints are found in the plastomes of all four Geraniaceae genera [24]. The simple relationship between chloroplast size and kmer diversity reveals the unique and highly variable nature of chloroplast evolution in the Geraniaceae, in comparison to other flowering plants.

We have six parasitic plants in the analysis, including two hemi-parasitic and two holo-parasitic species in the same genus (*Cuscuta*). The two pairs of species come together in this analysis according to their different levels of parasitism and all four fit the general trend discussed above where 18% of the chloroplast genome is duplicated in the IR regions. Two other unrelated holo-parasitic species (*Epifagus virginiana* and *Rhizanthella gardneri*: see *Ev* and *Rg* in Fig. 2A) have even more substantially reduced genome sizes and only *Epifagus* (*Ev*) departed from the general trend. This remarkable conservation of the ratio of single to double copy regions even after complete loss of photosynthetic function suggests that some other properties may be responsible for its stability. Other notable species include the subterranean clover, *Trifolium subterraneum* (*Ts* in Figure S2), is rather idiosyncratic among the legumes, as it lies between the 0–18% rate of duplication, while all other legumes tightly fit one or the other relationship. This intermediate level of diversity indicates an unusually high level of redundancy in the chloroplast genome, given the loss of the IR region,which has not been observed before.

Among more basal and deeply divergent genomes with two IR regions, two of the lower plants (*Tortula ruralis* - a moss [25] and *Huperzia lucidula*- a lycophyte [26]) and one basal angiosperm (*Illicium oligandrum*- star anise [27]) possessed significantly greater *k*mer diversity than would be expected, given the general trend line, indicating a lower sequence duplication rate than other chloroplasts with two IR regions. The vegetatively desiccation-tolerant moss, *Syntricha ruralis*, has both IR regions and, similar to the pines, has a rather small genome with a high *k*mer diversity. The basal angiosperm, *Illicium oligandrum* (*Io* in Fig. 2B), had roughly equivalent kmer diversity in relation to genome size as to clover. But the two species had achieved this relationship through very different mechanisms, with *Illicium* having lost roughly 1 kb in one copy of the IR region [27] reducing several typically duplicated genes to a single copy. These results indicate that the ratio of single to double copy nucleotide sequence in these lower plants is generally different than among the seed plants.

### Assembly properties of localized *de novo* contigs

**Figure 3:**
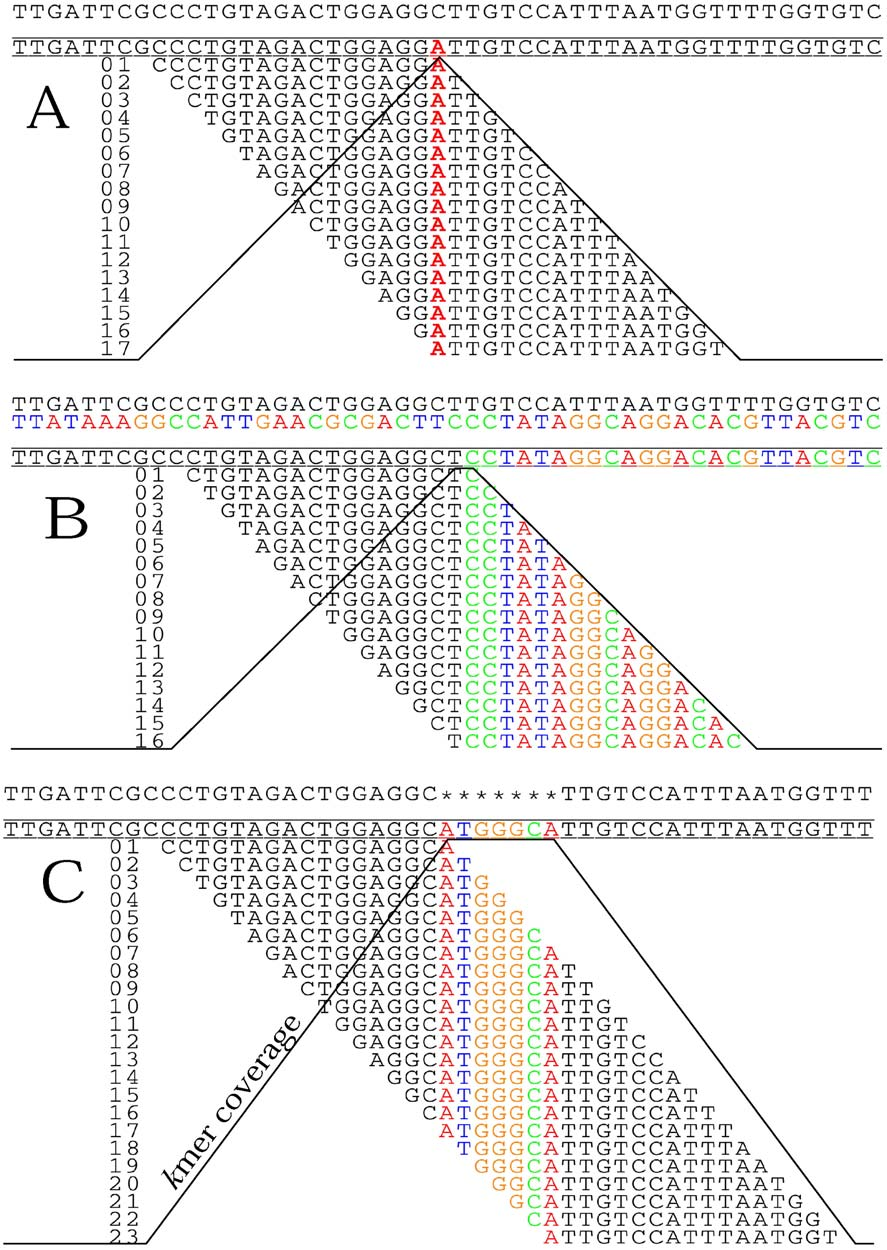
Distribution of informative *kmer*s possessing different types of polymorphisms, given *k* = 17. The query sequence is bracketed by two lines with the reference sequences above it. The *kmer*s containing the sequence variant are shown below. The black line indicates the coverage of the *kmer*s containing the polymorphism at each base position. A) single nucleotide polymorphism; B) translocation; C) insertion-deletion.

Different types of informative sequence variation will generate *de novo* contigs of different lengths, based on their *k*mer profiles (Fig. 3). If the variant involves a single nucleotide, the number of associated *k*mers will equal *k*, while a translocation or inversion event, resulting in a novel juxtaposition of sequence, *k*−1 fragments will contain the junction. In comparison to a single nucleotide, where coverage reaches a maximum at the position of the unique base, a translocation generates a two base plateau with maximum *k*mer coverage (Fig. 3B). An insertion-deletion event in one genome will have *k*+*i*−1 related kmers, where *i* is the length of the insertion (Fig. 3C). The *k*mer coverage profile will reach a maximum of *k* for the length of the insertion. These informative *k*mers will then select associated reads (Fig. 1 – Step 3), which will assemble into contigs centered on sequence variants.

**Figure 4:**
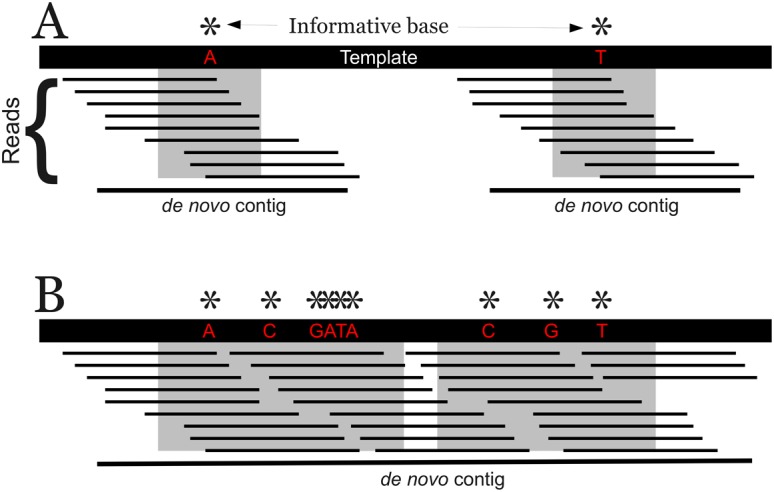
The relationship between local density of polymorphisms and the length of *de novo* contigs assembled from the reads possessing them. Each polymorphism is represented by an asterisk and the gray block indicates the window covered by the *kmer*s possessing the polymorphism, as shown in Fig. 3. The reads below each polymorphism contain a *k*mer that possesses the polymorphism. A) If local density of polymorphisms is low and the reads containing each polymorphism do not overlap significantly, then separate short contigs will be assembled, each containing a single polymorphism. B) If local density of polymorphisms is high, then reads will overlap substantially and even occasionally contain two polymorphisms, thus generating a single *de novo* contig for the entire region.

Generally, the majority of localized *de novo* contigs will contain a single feature and be relatively short (∼2*read length). Because most popular *de novo* assembly algorithms, like ABYSS used here, trim the low coverage ends from the contigs, trimming effects cause the actual lengths of contigs containing a single feature to be slightly shorter than two times read length [28]. If the genome has a very close relative in the analysis, then all of the contigs will be short, because single feature variants are will be scattered sparsely across the genome (Fig. 4A). If a region has a high density of informative sequence variation that falls within a window of roughly 2*read length, longer contigs will assemble (Fig. 4B). Due to the overlapping nature of the reads, the individual features are joined together in a single contig. If two very distantly related genomes are compared, the contigs distinguishing the two genomes will be quite long because of the accumulation of genetic differences and the increasing frequency with which a single read would contain two variants. The same would be true if a genome has undergone rapid diversification, particularly in a small region. Long contigs would be generated from these hotspots of nucleotide change. The size distribution of the localized *de novo* contigs in relation to the nearest relative in the analysis and mapping the contigs back to their reference genomes supports this idea.

The resulting assemblies can be separated into two major groups: “tip contigs” which represent assemblies for each genome based on nucleotide variation unique to that genome and “group contigs” which represent assemblies based on nucleotide variation shared by at least two and up to all of the genomes. We will first discuss some of the interesting patterns observed among the tip contigs and then cover the group contigs mentioned in the introduction.

### Tip contigs

Relatively few tip contigs, all mostly short and containing a ‘single feature’, were generated by genomes with two or more species in the same genus in the analysis (Table S2 – all tip contigs over 80 base pairs for each genome). Several taxa, including *Oryza sativa* v. *indica*, *Pinus gerardiana*, *Phyllostachys edulis* and both species of *Nicotiana*, produced only a small number of short tip contigs, indicating their limited divergence from their nearest relatives (NRs). The two longest tip contigs assembled (29,488 and 21,434 base pairs long) were both from *Adiantum capillusveneris* (maidenhair fern), which diverged from its most recent common ancestor (MRCA)with *Pteridium aquilinum* (common bracken) over 140 mya [13]. *Pteridium* also produced one tip contig over 20 kbp. Several other pairs of lower plants have substantially greater divergence times from their NR than these two ferns, including *Aneura mirabilis* (parasitic liverwort) and *Ptilidium pulcherrimum* (Naugehyde liverwort) that split over 300 mya; *Huperzia lucidula* (shining clubmoss) and *Isoetes flaccida* (quillwort) are separated by more than 380 mya. These deeper divergence times did not produce substantially longer contigs (a few >20 kbp), demonstrating that while phylogenetic signal did exist in the contig size distribution, tip contig length was not directly proportional to divergence time.

The two algae, *Chlamydomonas* and *Bryopsis*, represent the deepest split in the analysis, with an estimated divergence time greater than 700 mya but the longest tip contig produced by *C. reinhardtii* was only 6301 bp while *B. hypnoides* produced several longer contigs, including one >16 kbp. The shorter tip contigs for the former taxon may have been caused by the genomic expansion and proliferation of short repeats, on the scale of the value of *k* as discussed above. These types of repeats are known to cause loops in the *de Bruijn* graph and resulting in poor assembly. None of the 200 tip contigs produced by this alga, covering 86% (175 kb) of the genome, were significantly long, given the deep split with its nearest relative (NR). Over half of the tip contigs (106) were also ‘novel’ to the *Chlamydomonas* genome, not matching fragments from other genomes (BLAST evalue threshold <1e-10). These novel contigs were predominantly short, totaling less than 40 kb. Additionally, the super-abundant kmers were not present in the tip contigs. Because of the large number of possible results from this analysis, we will limit the remainder of our discussion to only significantly ‘long’ contigs. Long contigs were the longest 5% of the tip contigs produced by all taxa in each of six taxonomic classes (Figure S1). Additionally, if a long contig does not match any existing sequence in the NCBI database, other than itself (evalue threshold of 1̂-10 or less), then we considered the sequence ‘novel’.

### Within family level ‘long tip’ contigs

At the genus level, the two *Erodium* species each generated numerous long tip contigs, totaling over 50 kb and covering more than a third of each genome (Table 1). This result is remarkably different than all other genera with more than one species, indicating even more rapid evolution than in parasitic plants. As noted above, chloroplast evolution in the family Geraniaceae is obviously unusual [29], as each of the five species in the family produced a large amount of long tip contigs. While 6.8% of the contigs produced by one Erodium species were novel, none of the sequences from either *Pelargonium* or *Geranium* were novel. Therefore, the expansion of the very large chloroplast genome of *Pelargonium* [20] occurred without the *in situ* evolution or inter-genomic transfer of unique sequences. In the previous section, *Pelargonium* was extremely unusual for its very low *k*mer diversity compared to genome size but without any super-abundant *k*mers (Fig. 2), agreeing with the high level of repetitive DNA in repeats substantially longer than the value of *k*.

**Table 1:** Genomes with unusual tip contig characteristics.

			All tip contigs			Long tip contigs		Novel long tip contigs		
Family	taxa	No. kmers	No.	Length	% long	No.	Length	No.	Length	
Cupressaceae	*Cryptomeria japonica*	84827	264	117786	0.41156	28	48476	0	0	0
Geraniaceae	*Pelargonium x*	95177	276	117465	0.45651	31	53624	0	0	0
Cupressaceae	*Taiwania cryptomerioides*	83567	286	116096	0.375913	29	43642	3	3571	0.081825
Geraniaceae	*Geranium palmatum*	91352	356	115490	0.310365	20	35844	0	0	0
Geraniaceae	*Monsonia speciosa*	82439	224	110574	0.48761	31	53917	2	3774	0.069996
Fabaceae	*Lotus_japonicus*	74443	258	109227	0.611058	57	66744	0	0	0
Ranunculaceae	*Ranunculus macranthus*	70550	297	106331	0.314603	20	33452	0	0	0
Fabaceae	*Trifolium subterraneum*	76934	379	105686	0.355364	37	37557	1	803	0.021381
Geraniaceae	*Erodium texanum*	75326	327	105189	0.56195	69	59111	7	4003	0.06772
Fabaceae	*Cicer arietinum*	67412	311	102867	0.415196	42	42710	0	0	0
Geraniaceae	*Erodium carvifolium*	64479	277	98045	0.594135	63	58252	3	1596	0.027398
Orchidaceae	*Phalaenopsis aphrodite*	62810	300	96943	0.437061	39	42370	0	0	0
Fabaceae	*Glycine max*	60703	305	95779	0.459798	42	44039	0	0	0
Fabaceae	*Medicago truncatula*	59437	328	93636	0.303954	24	28461	0	0	0
Oncidiinae	*Oncidium Gower*	59206	313	93326	0.426366	33	39791	0	0	0
Fabaceae	*Lathyrus sativus*	59083	291	92427	0.381047	33	35219	2	1519	0.04313
Araceae	*Lemna minor*	57383	342	92378	0.315194	29	29117	0	0	0
Fabaceae	*Pisum sativum*	56662	317	91470	0.320619	24	29327	1	807	0.027517
Araceae	*Wolffiella lingulata*	58044	339	91023	0.347912	31	31668	4	3665	0.115732
Araceae	*Wolffia australiana*	57511	333	90593	0.345325	29	31284	1	760	0.024294
Araceae	*Spirodela polyrhiza*	54533	313	86740	0.32677	29	28344	1	1320	0.046571
Nymphaeaceae	*Nymphaea alba*	49327	347	85754	0.138746	10	11898	2	2588	0.217516
Nymphaeaceae	*Nuphar advena*	47769	329	82884	0.098777	6	8187	2	2166	0.264566
Fabaceae	*Vigna radiata*	50914	300	82511	0.358486	30	29579	0	0	0
Cuscuteae	*Cuscuta exaltata*	53811	239	81815	0.608409	52	49777	2	1257	0.025253
Cuscuteae	*Cuscuta reflexa*	51712	254	80748	0.553686	49	44709	4	2695	0.060279
Fabaceae	*Phaseolus vulgaris*	49394	292	80033	0.339298	28	27155	0	0	0
Apiaceae	*Daucus carota*	37308	322	67374	0.213376	17	14376	2	1671	0.116235
Euphorbiaceae	*Manihot esculenta*	35699	347	67328	0.144665	10	9740	2	1691	0.173614
Cuscuteae	*Cuscuta gronovii*	37430	220	55367	0.436903	36	24190	16	10171	0.420463
Cuscuteae	*Cuscuta obtusiflora*	36563	218	53901	0.443684	36	23915	13	8633	0.360987
Onagraceae	*Oenothera glazioviana*	6696	105	11182	0.079145	1	885	1	885	1

Tip contigs are assembled from the reads containing kmers unique to each genome.

Long tip contigs represent the top 5% of all local de novo contigs assembled in length.

Novel tip contigs did not match (e value ≤1̂-10) any existing sequence in NCBI other than itself.

The diversification of the chloroplast genome in all nine species of the legume (Fabaceae) family is obviously rapid and concentrated in hot-spots (Table 1), as each species produced >20 kb of ‘hot-spot’ contigs or at least 15% of the genome. *Lotus japonicus* (a small leguminous tree) produced >66 kb of these contigs, covering almost half of the chloroplast, but none of these sequences were novel (Table 1), which is considerably different than *Trifolium subterraneum* (subterranean clover), which also produced over 100 kb of tip contigs but only 38 kb were significantly long. Out of the 317 long tip contigs formed by the members of the legume family, only four were novel fragments. The presence in the NCBI database of a large amount of legume genomic sequence makes this result not so surprising and probably indicates that nuclear material has been repeatedly introduced into the legume chloroplast. BLAST results indicate that some fraction of these hot-spots closely match nuclear genomic sequences from model legume species, like *Medicago truncatula* and *Glycine max*.

The diversification of the chloroplast genomes obviously differed among families, as none of the 23 grass (Poaceae), 14 pine (Pinaceae), and 12 mustard (Brassicaceae) species were among the top 25 species for either length or novelty of contig sequence (Table 1), in sharp contrast to the geraniums, legumes, and parasitic *Cuscuta*. Several other plant families with at least five species in the comparison, including the Apiaceae, Asteraceae, Convolvulaceae, Onagraceae, and Solanaceae, were also poorly represented among the top 25 species for long contig length and novelty. The genomes of the two *Acorus* species were almost identical, given the small number of tip contigs assembled for either one.

Occasionally, the results were asymmetrical among several closely related species. Within the genus *Oenothera*, for example, two species (*O. biennis* and *O. elata*) produced half the tip contig length as the other three species (*O. argillicola*, *O. glazioviana*, and *O. parviflora*). Additionally, *O. glazioviana* produced one novel long contig that was 885 bases long. In the genus of parasitic dodder (*Cuscuta*), the four species were clearly separated into two groups, as the total length of long contigs for the two holo-parasitic species (*C. exaltata* and *C. reflexa*) was more than twice as much as for the two hemi-parasitic species (*C. gronovii* and *C. obtusiflora*), supporting the assumption that losing photosynthetic function accelerates sequence level divergence. Conversely, over half of the hemi-parasitic long contigs were novel (evalue <1̂-10 with existing sequence of other taxa in NCBI) sequences, a much larger proportion than in the holo-parasitic species, indicating that while nucleotide evolution accelerates, the introduction of novel sequence into the degrading chloroplast declines, as these *Cuscuta* species lost their photosynthetic function.

### Group contigs

The shared *k*mer (k = 21 bp) frequency table (Fig. 1 – step 2) contained almost two and an half million shared *k*mers. Only 214 out of the possible 174,391 groups shared equal to or more than 1000 *k*mers. These 214 groups shared over half of the *k*mers in the table, indicating strong signal in the data but also a lot of noise as well, as over one million of the shared kmers were simple homoplasy. Using the taxonomic code provided by NCBI for these 174 taxa, the possible number of groups that are taxonomically congruent is only 93. Viridiplantae, the group uniting all 174 taxa in the analysis, only shared 17 *k*mers and the reads pulled out of each genome by these shared *k*mers did not assemble, supporting the random nature of these shared *k*mers at such great phylogenetic distance. Additionally, twenty-one of the taxonomically congruent groups did not share more than 100 *k*mers (Table 2). Most of these ‘missing’ groups include large groups of genomes, ranging from orders to classes. The deeply divergent species in these groups would not be expected to share large pieces of identical nucleotide sequence. As discussed above, many of the divergences are considerably greater than 300 mya, including the two algae (Chlorophyta) and two primitive sporophytes (Isoetopsida).

**Table 2:** Characteristics of long group groups in deep taxonomic groups and big families.

Group	No. members	No. shared regions	Top regions
**Deeply conserved**			
magnoliids	5	43	exon, ycf2, rpoB
basalMagnoliophyta	5	42	ycf2, exon, rrn23
lamiids	12	28	exon, ycf2, rrn23
fabids	22	3	exon, rrn23, rrn16
asterids	24	8	exon, rrn23, ndhB
malvids	30	4	exon, rrn23, rrn16
rosids	53	2	rrn23, exon
coreeudicotyledons	79	1	rrn23
eudicotyledons	86	1	rrn23
**Big Families**			
***Fabaceae***			
Fabeae	2	88	exon, ycf2, rpoC2
Trifolieae	2	101	exon, ycf2, rpoB
Phaseoleae	3	86	exon, ycf2, rpoC2
Papilionoideae	9	9	exon, rrn23, rrn16
***Poaceae***			
Loliinae	2	53	rpoC2, exon, psbB
Triticeae	2	49	exon, exon_trnS-GCU, exon_rpl2
Poeae	3	88	exon, rpoC2, rrn23
Oryza	3	23	exon, rpoC2, rpl2
Andropogoneae	4	31	exon, rpoC2, exon_ycf3
Panicoideae	5	64	exon, rpoC2, ndhF
Pooideae	6	75	exon, rpoC2, psaA
Bambuseae	8	30	exon, ndhF, rpoC2
BEP_clade	17	47	exon, rpoB, psaB
Poaceae	23	29	exon, rrn23, rrn16
***Pinaceae***			
Pinus	8	71	exon, ycf2, rpoC2
Pinaceae	14	18	exon, rpoB, rpoC2
***Brassicaceae***			
Aethionema	2	60	exon, ndhH, ndhF
Arabideae	2	70	exon, ndhF, ycf2
Cardamineae	2	62	exon, rpoC2, matK
Camelineae	3	82	exon, rpoC2, ndhF
Brassicaceae	12	52	exon, ycf2, rpoB
**Top groups**			
Acorus	2	39	ycf2, ycf1, exon
Oenothera	5	23	exon, ycf2, ycf1
Eucalyptus	2	14	exon, exon_ycf3, exon_trnS-GCU
Populus	2	24	exon, exon_ycf3, rpl2
Nelumbo	2	26	ycf1, exon, exon_trnV-UAC
Gossypium	3	26	exon, exon_ycf3, exon_rps12_rps12_3end
Olea	2	16	ycf2, ndhF_ycf1, exon_ycf15_ycf2
Oryza	3	23	exon, rpoC2, rpl2
Cucumis	2	47	exon, ycf2, accD
Nymphaeaceae	2	69	exon, rpoC2, ndhF

Several groups of closely-related species at or above the family level were highly divergent and shared only a few *k*mers: included the four parasitic species of dodder (*Cuscuta*), the nine species of legume in the subfamily Papilionoideae, the three orchid (Orchidaceae) species, and the five species of morning glory (Convolvulaceae). All of these groups, except the legumes, involve parasitic plants, which have lost some, if not all, photosynthetic function in their chloroplast, again demonstrating the accelerated rate of evolution at the nucleotide level when selection pressures are relaxed. For legumes, the Papilionoideae subfamily is the only representative of the three major subfamilies in the Fabaceae but very few group contigs assembled at this level. In relation to the three other species-rich plant families in the analysis (12 cabbages - Brassicaceae, 14 pines - Pinaceae, and 23 grasses - Poaceae), the rapid evolution of the chloroplast genome in the nine legumes appears to be unique, as the other families share substantially larger numbers of *k*mers. Several legumes have lost one copy of the inverted repeat region and have undergone accelerated rates of structural change [30]. In general, the divergence times among the legumes are greater than those in these other families, but even the most divergent members of these families, which are generally more divergent in time, did not generate considerable amount of tip contigs.

Given these broad patterns and the very large number of possible comparisons, we will only directly address two major comparative questions among the group contigs: 1) what chloroplast regions are deeply conserved among deep groups in the flowering plants; and 2) does chloroplast evolution differ among the four species-rich families in our analysis: the legumes (9 species), the grasses (23), the mustards (12), and the pines (14). As for the results for tip contigs, we limit our analysis to long contigs produced using the data with error. These long contigs are certainly not the only informative ones in the analysis because the numerous short contigs each contain a single informative feature. Our focus on the long contigs is merely a way to limit the discussion for the purposes of this manuscript. All of the contigs, long and short, are provided in the supplementary material (Table S3). For validation and accuracy purposes, all of these long contigs were mapped back to their reference genomes and the associated gene map produced by DOGMA [31]. The contigs were highly accurate with >97% having 100% identity with their reference sequence, even though we introduced a 3% error rate while simulating the short reads sequencing. All of the contigs produced for the taxonomically congruent groups, as analyzed here, are available for download (Table S3).

### Deeply conserved elements

To reveal the chloroplast elements that are the most deeply conserved among flowering plants, we assembled ‘group’ contigs for the following taxonomic groups, as defined by NCBI (see Table S1): eudicots (86 species), core eudicots (79), basal Magnoliophyta (5), asterids (24), fabids (22), lamiids (12), magnoliids (5), malvids (30), and rosids (53). These ‘group’ contigs are assembled from the reads which contain the kmers that are shared by all members of the group (see Fig. 1). Because the primary function of the chloroplast is photosynthesis and elements associated with this function would most likely be deeply conserved, we removed the parasitic plants from this analysis, particularly because they were so clearly divergent at the nucleotide sequence level in our previous results, even at the family level.

At the level of the eudicots (the monophyletic dicotyledonous plants, excluding the basal Magnoliophyta) and the core eudicots, a ∼300 base pair portion of the *rrn*23 gene (see Table S3), which codes for 23S ribosomal RNA, was the longest *de novo* contig shared by all 86 species (Table 2) and this conserved sequence is duplicated in the IR regions. More detailed analysis revealed that two copies of these sequences can still be found in all legume species that have lost one copy of the IR region (see SOM 3). Unlike other gymnosperms with only one IR region, the fragment is also duplicated in *Cryptomeria japonica*, suggesting that two copies of this core element remains essential despite the loss of the second IR region. Notably, this fragment is not duplicated in the other gymnosperms with only one IR region. *Monsonia speciosa* possesses the only land plant chloroplast without the entire rRNA operon [32] and yet it still shares this deeply conserved portion of the *rrn*23 gene. The rosids (53 species) shared an additional portion of this gene region.

The magnoliids, the basal Magnoliophyta, and the lamiids produced the greatest number and longest total length of long group contigs (40 kb, 28 kb, and 25 kb/species, respectively), which is certainly related to the fact that these are also the smallest groups in the analysis. The *ycf*2 gene was consistently covered by the greatest number of long contigs in these three groups, while the magnoliids shared a portion of the *rpo*B gene (Table 2). The asterids (sunflowers and their relatives) produced twice as much shared sequence in long contigs as the fabids (legumes and their relatives) and the fabid group shared considerably fewer regions among its members than the asterid group, despite the fact that the asterids has more species in the group than the fabids. Increasing numbers of members in the group would typically be associated with declining amount of shared sequence among all of the members of the group. These results indicate that the chloroplast genome in the sunflowers is more conserved and stable at the nucleotide level. The asterids were also unusual as conserved regions of the *ndh*B gene were shared by all 24 species. This gene was not shared by any other group of taxa analyzed. All the remaining groups also shared intronic regions, which remain uncharacterized but may serve an equally important but unidentified chloroplast function. More detailed and specific research on each group is required to begin to understand the importance of these regions.

### Comparative genomics of four families

Four families with numerous species in the analysis (legumes: 9; mustards: 12; pines: 14; grasses: 23) with substantially different estimates for divergence times at the family level, from 170 mya for the pines and 55 mya for both the legumes and mustards. The number of group contigs for both infra-familial and familial level comparisons and the genic regions the long contigs were associated with varied considerably among these four families (Table 2). While the three tribes of legume (Fabeae, Trifolieae, and Phaseoleae) shared the largest number of long group contigs (>80), than almost any other group, this high degree of similarity breaks down quickly at the subfamily level. When all nine species are included, only nine long contigs are generated. On the other hand, the several subfamilies of grasses and several large clades, including the BEP clade [33] with seventeen species and the entire family with 23 species, shared many more long group contigs (Table 2). Likewise, the twelve species of mustard (Brassicaceae) in the analysis shared over 50 long group contigs. In gymnosperms, the eight species of *Pinus* share a considerable number of long contigs, while at the family level, with 14 species, the assembly is considerably smaller than either mustards or grasses.

These patterns indicate that a dramatic change at the nucleotide level had occurred among the various tribes of the legumes, within which chloroplast structure has been relatively conserved, but all of the mustard and the grass species retain a much larger fraction of the ancestral chloroplast sequence. Additionally, while the chloroplast genomes of *Pinus* species have a relatively conservative sequence, the various genera of the Pinaceae are strongly divergent, only producing 18 long contigs. Finally, the three tribes of grasses (Loliinea, Triticeae, and Poeae) share fewer group contigs than the tribes of legumes because each set of group contigs is assembled from the shared kmers unique to each group (Fig. 1). Therefore, if all species of the family share a large fraction of their genomes, these shared kmers cannot also be shared uniquely by two species in a single tribe. The sets of group contigs are not strictly nested, although they can possibly overlap.

Because the genes on the chloroplast are well-characterized and an automated protocol for annotating plastid genomes exists [31], we can compare the shared regions among the various groups to examine how these differences might relate to function. Each of the three tribes of legumes share substantial but distinct portions of the *ycf*2 gene (Table 2), indicating that this gene may have played a significant role in its diversification. The profound nature of the divergence in chloroplast sequence among the legume subfamilies is also highlighted by the fact that among the few long group contigs shared by all nine legume species, these are largely confined to portions of the highly conserved and essential ribosomal genes (*rrn*23 and *rrn*16). The four tribes in the mustard family (Aethionema, Arabideae, Cardamineae, and Camelineae), each of which contain 2–3 species, shared between 60–82 regions and the *ndh*F gene was commonly shared although the other important shared regions varied considerably among these groups. When all twelve species in the mustard family are examined, the *ycf*2 gene was an important shared element.

The grasses, the largest family represented among reference chloroplast genomes, also have the most detailed and complicated subfamilial taxonomic structure (Table 2). These groups range from the genus up to large groups defined by specifics of their physiology and most of these groups shared a large number of regions, even at the family level as noted above. The *rpo*C2 gene, responsible for synthesizing RNA polymerase beta chain, is frequently the most common shared region among the smaller taxonomic groups, particularly in Loliinae, one of the few examples where the intronic regions are not the most commonly shared region. The grasses are also unusual because several smaller taxonomic groups share junctions between intronic and genic regions, including the junction with a *trn* gene, the only example of these abundantly studied genes among the most covered shared regions. The most covered shared regions among the Triticeae group were both junctions between intronic and genic regions. While the grasses demonstrated a fairly consistent pattern of shared regions, both in quantity and specific regions, a diverse set of genes were the third most covered region among the smaller taxonomic groups.

Obviously, for other genomic compartments, like the nucleus, or in poorly studied organisms, our reference-free approach would not allow direct analysis of the function of the shared *de novo* contigs. Here, we are simultaneously demonstrating the power of the approach and validating the results, by referring back to previous findings in the literature and the existing functional annotations. In this section on ‘group’ contigs, we chose to focus on a very small subset of the overall results of the analysis, discussing on the deeply conserved elements and the differences among the four large families present in the analysis. A large number of additional findings could be quickly extracted for specific taxa and groups. We can provide the full shared kmer table for all 174 taxa and any set of localized *de novo k*mers, reads, or contigs upon request.

## Conclusions

Our reference-free approach is an efficient and powerful way to compare unassembled short-read genomic sequence data. Here, we analyzed genomic sequence data from 174 plant chloroplasts, across a wide range of taxonomic relationships and divergence times, providing a broad perspective on chloroplast evolution in Viridiplantae and a rich framework for further exploration. Each step in our reference-free pipeline can produce significant results and can be analyzed in various ways, from the simple relationship between genome size and *k*mer diversity at the first step to size distribution of the localized *de novo* contigs. For example, we found that the ratio of DNA sequence duplicated in the Inverted Repeat regions of the green plant chloroplast was strongly conserved across a four-fold change in chloroplast genome sizes, from greatly reduced genomes in several non-photosynthetic parasites to greatly expanded genome in an alga. This ratio remained unchanged despite obvious differences in the types of repetitive elements, among some of these taxa. This evolutionary conservation of this molecular structure in species where photosynthetic function has been completely lost indicates the the mechanism maintaining this ratio of duplicated sequence cannot be related to photosynthesis. Instead other critical functions, related possibly to genomic replication, gene expression, or cytoplasmic identity, may be responsible, although we have no way of distinguishing among these functions.

Our results agreed with and extended previous studies of several taxa, including the Geraniaceae and the clade of legumes that have lost their inverted repeat. More importantly, the localized *de novo* assemblies for each tip genome and set of target groups examined in detail produced a rich framework of information about genomic differences and similarities. Given the statistical properties and the group membership of the localized contigs, we can extract either rapidly evolving or deeply conserved regions of the genome and associate these regions with existing annotation information via simple BLAST searches. Many genomic characteristics were lineage-specific, particularly at the family level among the grasses, legumes, and mustards, but a few extended to the split between gymnosperms and flowering plants. Our approach will be particularly useful for analyzing the increasing number of whole genomic short read sequencing (SRS) datasets from previously unstudied organisms, when whole genome assembly is difficult or low coverage data prevents adequate assembly. We strongly feel that the combination of high throughput SRS technologies and our reference-free analytical approach can greatly expand the scope of comparative genomics in biodiversity and conservation.

## Materials and Methods

### Analytical approach

Our current analytical pipeline extends and generalizes a previously published pipeline [1], improving its computational efficiency and downstream analysis. The pipeline involves four main steps (Fig. 1). The phylokmer package for generating the kmer frequency tables and python scripts to perform both the tip and group analyses on the resulting kmer tables are available for download at: http://sourceforge.net/projects/referencefree/.

- **Step One.** Generate frequency tables of all unique *k*mers for each set of genomic data. Each *k*mer and its reverse complement are equated, taking the one of the pair which comes first alphabetically. All unique *k*mers are sorted alphabetically in each genomic kmer table. The frequency of each kmer includes the sum of its occurrences in both directions. A frequency threshold can be set to screen out most random errors. We have typically found that a threshold of three is adequate to remove a substantial portion of these errors. We used a program written by J. Ruan called ‘kmer_count’ (part of the phylokmer package) to generate these frequency tables, although any valid kmer counter could be used.
- **Step Two.** Separate tables are generated for each genome containing the *k*mers observed to be unique for that genome and and a large table containing all of the *k*mers shared by at least two genomes (see python scripts). These tables are generated by a program called ‘filter’ written by J. Ruan. The tables containing the kmers unique to each genome are referred to as ‘tip *k*mers’ while those shared among genomes are referred to as ‘group *k*mers’. For example, in Figure 1, all *kmer*s present in both genomes A and C but not in any other genomes would be assigned to “group AC”. These *kmer*s would correspond to the green circle emerging from the shared kmer frequency table (Fig. 1 – Step 2). For the group analysis, two strategies can be pursued. In either strategy, a specific list of group comparisons can be supplied by the user to limit the total output. The first strategy assumes an ‘exclusive intersection’ was performed and the group kmers shared by the member genomes are ‘exclusive’ to those genomes and are absent from all other genomes. The second strategy assumes ‘inclusive intersection’ was performed and the group kmers shared by the member genomes could also be shared with other genomes, which are not members of the group. The second strategy assumes no knowledge about the other genomes. Using this strategy, only the pair-wise comparisons are necessary to capture all of the information, as the results can be intersected later to obtain the results for groups with more than two members. The first strategy is best when the results from only a small subset of possible groups is needed, while the second strategy provides a comprehensive and flexible result, although it can require larger amounts of storage, processing time, and bioinformatic expertise in handling the downstream analysis of larger groups.
- **Step Three.** Segregate the SRS reads that contain each subset of ‘tip’ or ‘group’ kmers into smaller datasets. For each member of a ‘group’, the reads from each genome are maintained separately. Here, we use a program written by C.X. Ye called “ReadsSelector’, which is included in the package.
- **Step Four.** Assemble the localized *de novo* contigs from each set of reads, relevant to the comparative question being asked. We assembled all tip contigs and all group contigs congruent with the accepted taxonomy of the taxa, according to the NCBI database (see Table S1). We used ABYSS [28] for our *de novo* assembly because it worked easily with our python scripts but any *de novo* assembly program could be used. Because the complexity of assembly is relatively simplified for each subset of reads, more comprehensive approaches for whole genome *de novo* assembly may provide better results.

Initially, we explored three different values of k (17, 21, and 25 base pairs) but we detected only trivial differences among the results for this range of k values. The results for this range of k values were also considerably more informative than much larger or much smaller values. Therefore, we only present results using k = 21. Several downstream analyses of the *k*mer and contig properties were written in Mathematica 7 by C.H. Cannon. The pipeline does not require a super-computing environment and can be run effectively on a dual-core laptop computer with at least 4 GB of RAM, although this is certainly proportional to the amount of raw data used.

### Genomic data and diversity

We downloaded 174 complete chloroplast genomes of Viridiplantae from NCBI (see Table S1 for complete list of accession numbers, basic information, and taxonomy) and simulated short read sequencing both with and without error, using dwgsim [12], assuming paired-end libraries, until we achieved roughly 20× coverage for each taxa. To examine the relationship between overall diversity of unique kmers and genome size for each chloroplast, we simply counted the number of kmers in each frequency table (from Step 1 above) and plotted this against the published genome size. A line was fit to the genomes with the IR regions present.

### ‘Tip’ and ‘group’ contigs

As stated in the introduction, we limited the analysis of the localized *de novo* contigs to the ‘long’ contigs. These contigs represent the longest 5% of the contigs in each tip and group analysis. Because of the extremely large number of possible groups, given 174 genomes, we limited our group analysis to those groups described in the introduction. These contigs were blasted against the nucleotide collection at NCBI and the relevant information extracted from the downloaded results.

## Supporting Information

Figure S1 Size distribution of tip contigs in six different taxonomic classes.
(TIF)

Figure S2 Relationship between genome size and kmer diversity, specifically for the angiosperms.
(TIF)

Table S1 This XLS-format spreadsheet file provides the relevant reference information about the 174 chloroplast genomes analyzed in this study, including NCBI accession numbers and basic genomic properties.
(XLS)

Table S2 This zipped archive includes a separate file for each of the 174 taxa analyzed in this study. Each file contains the tip contigs assembled for each genome that were greater than 80 base pairs in length, in FASTA format. The zipped archive can be downloaded from the following website: http://www.ecologicalevolution.org/content/2012/04/174taxa_tipcontigs_k21_n3.zip.
(ZIP)

Table S3 These are the group contig files assembled for the two large groups analysed in this study: A) “Deep groups” and B) “Big families” (see results and discussion). These two files contain all of the de novo group contigs assembled that were over 80 base pairs for each member of the group in FASTA format. The annotation line for each sequence contains the taxa name and relevant group information.
(DAT)
