# Supplementary figures and images for: Reference-Free Comparative Genomics of 174 Chloroplasts
Source: PLoS One. 2012 Nov 20;7(11):e48995. doi: 10.1371/journal.pone.0048995 (PMC3502452; doi:10.1371/journal.pone.0048995)

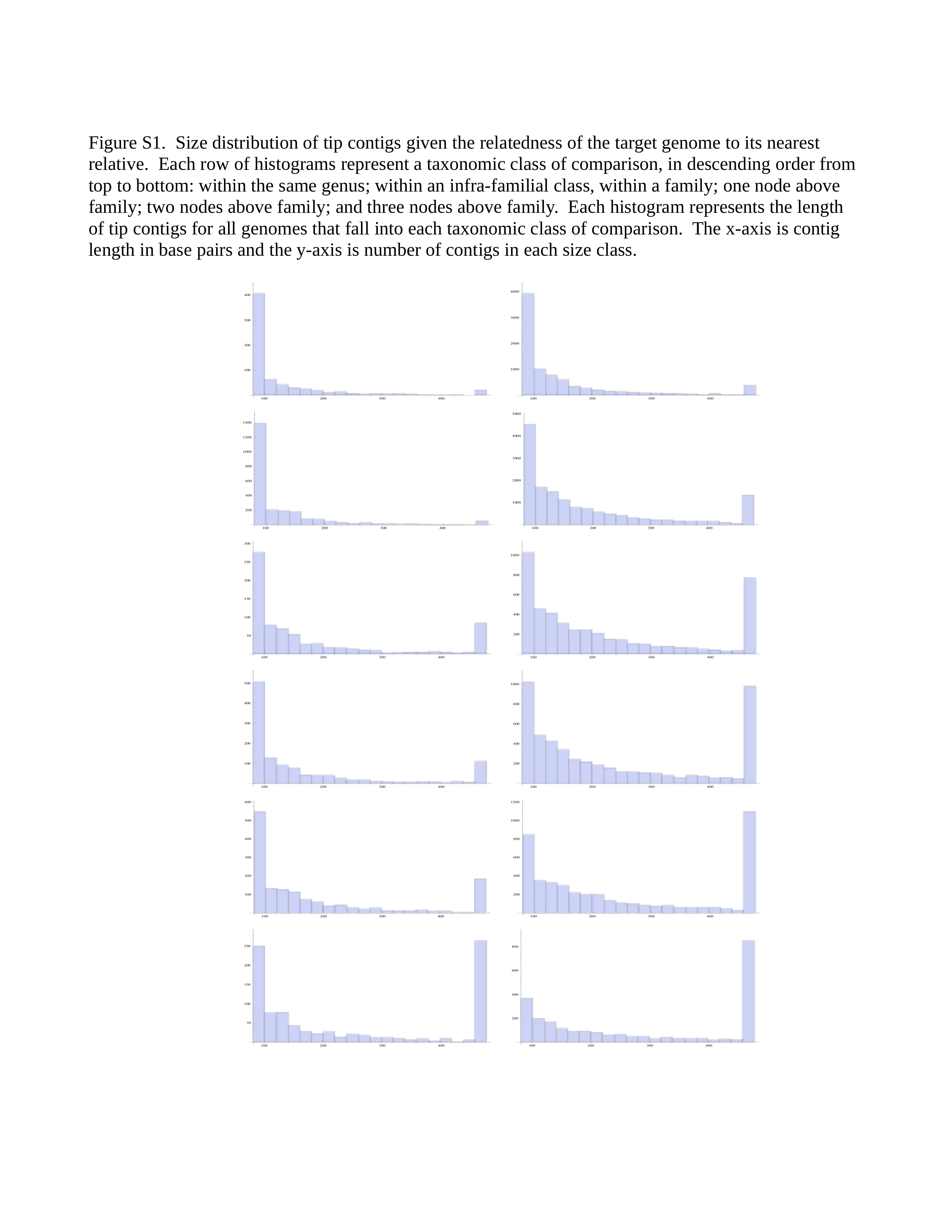

Supplement: Figure S1 — Size distribution of tip contigs in six different taxonomic classes. (TIF) [file pone.0048995.s001.tif]

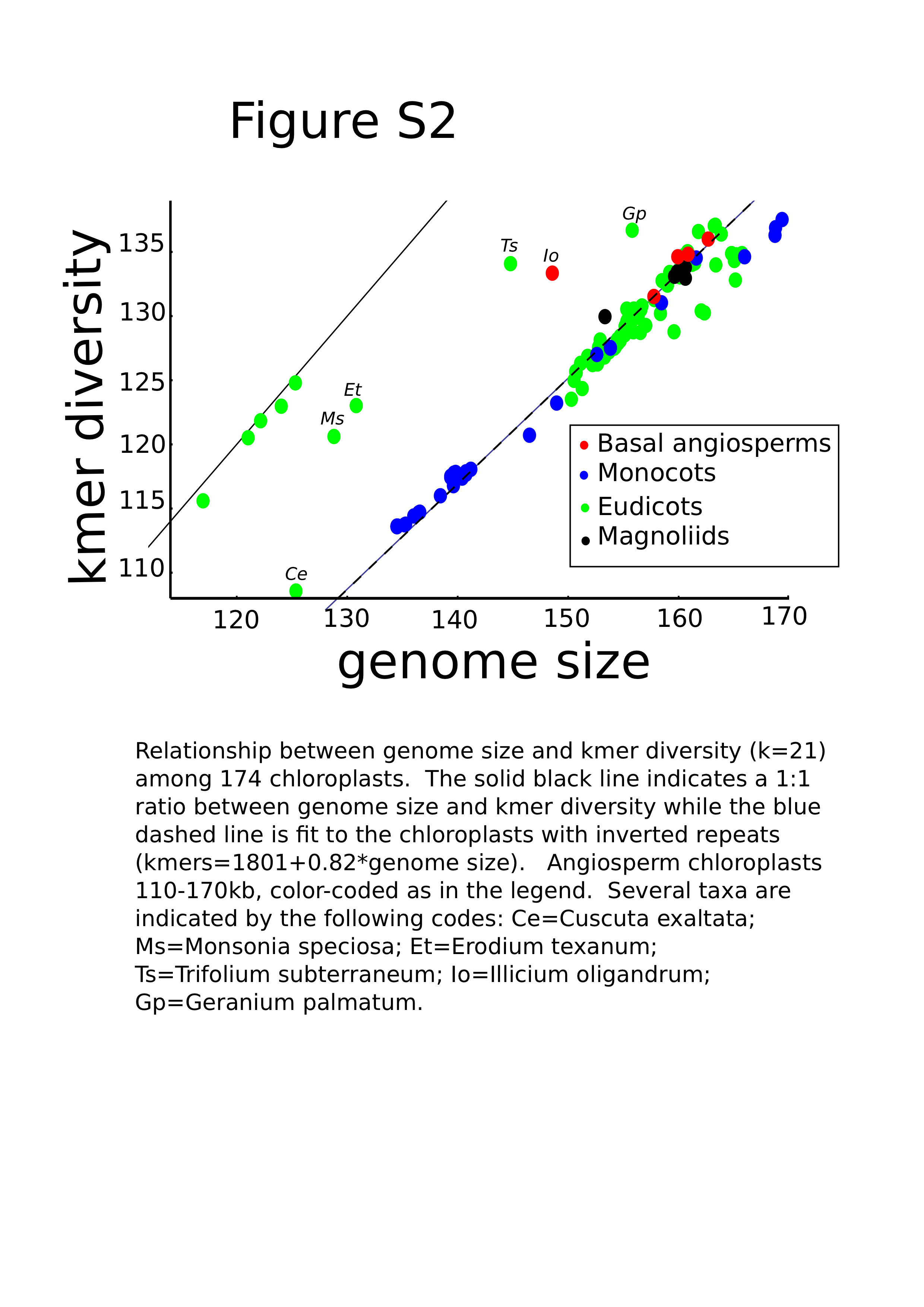

Supplement: Figure S2 — Relationship between genome size and kmer diversity, specifically for the angiosperms. (TIF) [file pone.0048995.s002.tif]
